# How to reduce cisatracurium consumption in ARDS patients: the TOF-ARDS study

**DOI:** 10.1186/s13613-017-0305-2

**Published:** 2017-08-02

**Authors:** Sami Hraiech, Jean-Marie Forel, Christophe Guervilly, Romain Rambaud, Samuel Lehingue, Mélanie Adda, Pierre Sylla, Sabine Valera, Julien Carvelli, Marc Gainnier, Laurent Papazian, Jérémy Bourenne

**Affiliations:** 1 APHM, URMITE UMR CNRS 7278, Hôpital Nord, Réanimation des Détresses Respiratoires et Infections Sévères, Aix-Marseille Univ, Marseille, France; 2 Réanimation des Urgences et Médicale, CHU la Timone 2 Marseille, Aix-Marseille Université, 13385 Marseille, France; 3 Réanimation– Détresses Respiratoires et Infections Sévères, CHU Nord, Chemin des Bourrely, 13015 Marseille, France

**Keywords:** ARDS, Neuromuscular blockers, Cisatracurium, Train-of-four, Cost

## Abstract

**Background:**

Neuromuscular blocking agents (NMBAs) have been shown to improve the outcome of the most severely hypoxemic, acute respiratory distress syndrome (ARDS) patients. However, the recommended dosage as well as the necessity of monitoring the neuromuscular block is unknown. We aimed to evaluate the efficiency of a nurse-directed protocol of NMBA administration based on a train-of-four (TOF) assessment to ensure a profound neuromuscular block and decrease cisatracurium consumption compared to an elevated and constant dose regimen. A prospective open labeled study was conducted in two medical intensive care units of two French university hospitals. Consecutive ARDS patients with a PaO_2_/FiO_2_ ratio less than 120 with a PEEP ≥5 cm H_2_O were included. Cisatracurium administration was driven by the nurses according to an algorithm based on TOF monitoring. The primary endpoint was cisatracurium consumption. The secondary endpoints included the quality of the neuromuscular block, the occurrence of adverse events, and the evolution of ventilatory and blood gas parameters.

**Results:**

Thirty patients were included. NMBAs were used for 54 ± 30 h. According to this new algorithm, the initial dosage of cisatracurium was 11.8 ± 2 mg/h, and the final dosage was 14 ± 4 mg/h, which was significantly lower than in the ACURASYS study protocol (37.5 mg/h with a constant infusion rate (*p* < 0.001). The overall cisatracurium dose used was 700 ± 470 mg in comparison with 2040 ± 1119 mg for patients had received the ACURASYS dosage for the same period (*p* < 0.001). A profound neuromuscular block (TOF = 0, twitches at the ulnar site) was obtained from the first hour in 70% of patients. Modification of the cisatracurium dosage was not performed from the beginning to the end of the study in 60% of patients. Patient–ventilator asynchronies occurred in 4 patients.

**Conclusion:**

A nurse-driven protocol based on TOF monitoring for NMBA administration in ARDS patients was able to decrease cisatracurium consumption without significantly affecting the quality of the neuromuscular block.

## Introduction

Despite continuous research, acute respiratory distress syndrome (ARDS) is still associated with significant mortality [1]. Only three therapeutic measures have been shown to improve the survival of ARDS patients in large randomized controlled trials (RCTs): the reduction in tidal volume to 6 mL/kg of predicted body weight (PBW) [2], a short course of neuromuscular blocking agents (NMBAs) [3] in severely hypoxemic patients, and the use of prone positioning (PP) [4]. NMBAs are frequently used in the most severe forms of ARDS [1]. They are the only pharmacologic intervention that has been shown to improve the prognosis of moderate-to-severe ARDS patients when used at the early phase and for a short duration [3]. Even if their mechanisms of action are uncertain, NMBAs help to ensure a protective lung ventilation at the acute phase of lung damage by limiting spontaneous breathing efforts and asynchronies [5, 6], preventing ventilator-induced lung injury [7, 8], and limiting plateau pressure, which decreases baro- and volutrauma and avoids lung derecruitment by abolishing expiratory efforts [9]. Recent data also showed that NMBAs cause an increase in the inspiratory and expiratory transpulmonary pressure [9], favoring lung recruitment. A proper anti-inflammatory effect has also been suggested [7, 8, 10].

Recent guidelines recommend the use of NMBAs in ARDS patients who have a PaO_2_/FiO_2_ ratio under 150 [11]. However, an accurate dosage of cisatracurium is currently not recommended. In the ACURASYS study [3], the investigators used a high and constant dosage of cisatracurium to achieve a profound paralysis considering the fact that there was no monitoring of the train-of-four (TOF) to ensure blindness. Consequently, the accurate posology of cisatracurium to use during ARDS is unknown. Recently, published data showed that ARDS patients are frequently insufficiently paralyzed when following former recommendations [12]. Even if high doses of NMBAs are necessary to paralyze the diaphragm, the ACURASYS posology used in all patients might, however, be excessive and associated with high costs. A reduction in cisatracurium use could limit “over-paralysis” and NMBA side-effects and decrease costs.

Sedation managed according to a nurse-driven protocol to obtain the lowest effective dose has been proven to reduce ICU mortality, length of mechanical ventilation (MV), and duration of ICU and hospital stay [18–20]. In 2004, Baumann et al. evaluated a nurse protocol of NMBA management for all the ICU patients requiring paralysis. They found no reduction in NMBA use in the protocol group compared to the control group [13]. Similar results were found from a study by Strange et al. [14] using atracurium. The authors concluded in a non-interest of monitoring a neuromuscular block in ICU patients. However, both studies did not specifically focus on ARDS patients. A recent study [12], including a large cohort of ARDS, emphasized the discrepancies between clinical judgment and TOF monitoring, showing that ARDS patients were frequently under-paralyzed, whereas clinicians considered that the neuromuscular block obtained was sufficient. These data strongly suggest that TOF monitoring is useful to objectively assess the neuromuscular block. However, no study has investigated the consumption of cisatracurium and the efficacy of the neuromuscular block when the administration is based on a nurse-driven protocol at the acute phase of ARDS.

Therefore, the aim of the present study was to determine whether a nurse-driven algorithm based on TOF monitoring in ARDS patients could help reduce the amount of cisatracurium administered compared with the ACURASYS study dose regimen without deleterious effects.

## Patients and methods

### Type of study

We conducted a prospective open study in two French medical ICUs of two university hospitals. The inclusion criteria included the following: patients with moderate-to-severe ARDS (PaO_2_/FiO_2_ ratio <120 with an applied PEEP of 5 cm H_2_O) requiring a continuous administration of NMBAs. The exclusion criteria included the following: patients less than 18 years of age, pregnant women, tetraplegia before ARDS, extra-corporeal membrane oxygenation (ECMO) or extra-corporeal CO_2_ removal (ECCO_2_R) requirement, and previous use of continuous cisatracurium treatment during the same ICU stay. The study protocol was approved by the Ethics Committee of the French Intensive Care Society (N°14-32).

### Study objectives and parameters

**Fig. 1:**
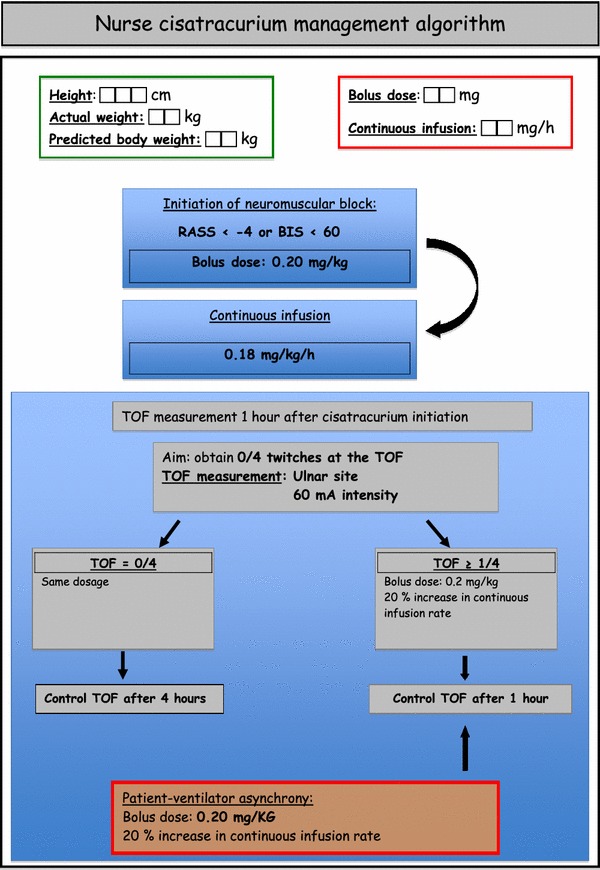
Nurse-directed protocol of TOF monitoring and cisatracurium management

All the patients included received a continuous infusion of cisatracurium with an initial dosage based on current guidelines [15]. Neuromuscular block monitoring and cisatracurium dosage modifications were achieved by nurses according to an algorithm (Fig. 1).

The main objective of the study was to compare the cumulated dosage of cisatracurium used to the dosage that patients would have received if they had been treated according to ACURASYS study protocol (i.e., 37.5 mg/h with a constant dosage and no monitoring of neuromuscular block).

The secondary objectives were to assess the effectiveness of neuromuscular block, the rate of dosage modifications of cisatracurium, the number of NMBA boluses needed, and the complications observed such as clinically detectable patient-to-ventilator asynchronies.

The parameters studied included demographic parameters, ARDS characteristics, patient care, ventilatory modalities, and other therapeutics used such as prone position or inhaled nitric oxide (iNO). Data were collected throughout the period of paralysis and until the fourth day.

### Mechanical ventilation

Patients were all managed throughout the study according to the original ARDS-net protocol [2]. Briefly, patients were ventilated in a volume-assisted controlled mode with constant square flow with a tidal volume of 6 mL/kg/PBW. The goal of oxygenation was to target a peripheral saturation of blood oxygen (SpO_2_) measured by pulse oximetry between 88 and 95% or a PaO_2_ of 55–80 mmHg measured by arterial blood gas analysis. To achieve this goal, FiO_2_ and positive end-expiratory pressure were adjusted as in the ARMA study [2]. Respiratory rate was adjusted to ensure an arterial pH range between 7.20 and 7.45. Ventilatory parameters and blood gas exchange were followed daily until day 4, even if cisatracurium infusion had been discontinued by physicians.

### Management of sedation and neuromuscular blocker administration

The Richmond Agitation-Sedation Scale (RASS) was used to adapt sedative requirements prior to starting NMBAs [16]. We used a continuous infusion of midazolam and sufentanil to achieve a RASS inferior to −4 [17] or a bispectral index under 60 if available [18]. If this goal was not achieved, a continuous infusion of ketamine was added. When the RASS objective was reached, nurse-directed NMBA administration was started.

Consecutive patients with ARDS who met the inclusion criteria were included in the study. The initiation, as well as the interruption of NMBA administration, was decided by the physician in charge of the patient. The patient received a 0.20 mg/kg rapid intravenous infusion of cisatracurium besylate, followed by a continuous infusion of 0.18 mg/kg per hour. The dosage of cisatracurium besylate was adjusted according to the patient’s PBW.

A peripheral nerve stimulation was performed 1 h after the beginning of the infusion. The objective of paralysis was to obtain a train-of-four (TOF) equal to zero twitches. The train-of-four was monitored on the *adductor pollicis* with a 60-mA intensity [19].

If the patient had no twitch, no modification of dosage was performed and TOF was monitored every 4 h.

If the patient had 1 or more responses, a supplementary bolus of cisatracurium (0.20 mg/kg) was administered and the dosage of the continuous infusion was increased by 20%. A new peripheral nerve stimulation was performed 1 h after the dosage increase. If the objective of paralysis was attended, the train-of-four was monitored every 4 h. A supplementary bolus of cisatracurium (0.20 mg/kg) was administered if patient–ventilator asynchrony was diagnosed. Nurses recorded the values of TOF, the modifications of cisatracurium dosage and the additional boluses performed. The total dosage of cisatracurium used was noted every 12 h. Clinically detectable patient–ventilator asynchronies and the occurrence of adverse events such as pneumothoraces or a decrease in SpO_2_ under 85% were also recorded.

### Statistical analysis

Continuous variables were expressed as the median and interquartile range and compared using Wilcoxon’s rank-sum test. The *χ* ^2^ test or the Fisher exact test was used to compare categorical variables. Multivariate logistic regression was used to identify the independent factors associated with increased NMBAs dosages. The Hosmer–Lemeshow test with a *p* >0.05 suggests a good fit between data and the logistic regression model. All variables that exhibited a *p* value <0.2 on univariate analysis were entered in the model. Interactions were tested in the model; variables strongly associated with other(s) were not included in the multivariate analysis. A two-tailed *p* ≤0.05 was considered statistically significant. Statistics and figures were performed with SPSS 20.0 (SPSS Inc. Chicago, IL).

## Results

### General characteristics

Thirty patients were prospectively included in this study. The primary characteristics at inclusion are summarized in Table 1. The main etiology of ARDS was pneumonia. Eighteen patients (60%) had moderate ARDS and the remaining 12 patients presented with severe ARDS. Table 2 summarizes the evolution of ventilatory parameters and blood gas exchanges during the study period. Thirteen patients (43%) were prone positioned, and 6 (20%) received inhaled NO for hypoxemia.

**Table 1:** Patient characteristics at the time of inclusion

Characteristics	
Number of patients	30
Age (years) mean ± SD	60 ± 16
SAPS 2 score median (IQR)	46 (38–53.5)
SOFA score median (IQR)	8 (7–10)
Etiology of ARDS no./total no. (%)	Community acquired pneumonia 11/30 (37)
	Ventilator associated pneumonia 10/30 (33)
	Aspiration pneumonia 6/30 (20)
	Extra pulmonary ARDS 3/30 (10)
Topography of ARDS no./total no. (%)	Lobar ARDS 13/30 (43)
Diffuse ARDS 17/30 (57)	
PaO_2_/FiO_2_ ratio median (IQR)	102 (77–120)
Vt (mL) ± SD	385 ± 76
Vt mL/kg (PBW)	6.1 ± 1.1
Pplat median (IQR)	25 (22–27)
PEEP median (IQR)	10 (8–14)
FiO_2_ median (IQR)	75 (60–100)
Other conditions no./total no. (%)	Septic shock 27/30 (90)
	Renal replacement therapy 1/30 (3)

**Table 2:** Evolution of ventilatory parameters and blood gas exchange from day 1 to day 4

Days after inclusion	Day 1	Day 2	Day 3	Day 4
Number of patients	30	28	27	27
Vt (mL) ± SD	385 ± 76	384 ± 62	387 ± 68	387 ± 47
Vt mL/kg (IBW)	6.1 ± 1.1	6.1 ± 0.9	6.2 ± 1.2	6.2 ± 0.7
PaO_2_/FiO_2_ ratio median (IQR)	102 (77–120)	143 (112–207)	161 (132–239)	154 (134–227)
Pplat median (IQR)	25 (22–27)	25 (23–27)	26 (24–27)	26 (23–27)
Driving pressure cm H_2_O median (IQR)	13 (11–16)	13 (11–15)	14 (12–16)	14 (10–16)

### NMBA consumption

The average duration of NMBA use was 54 ± 30 h. The mean initial dosage of cisatracurium was 11.8 ± 2.0 mg/h, and the mean final dosage was 14 ± 4 mg/h.

**Fig. 2:**
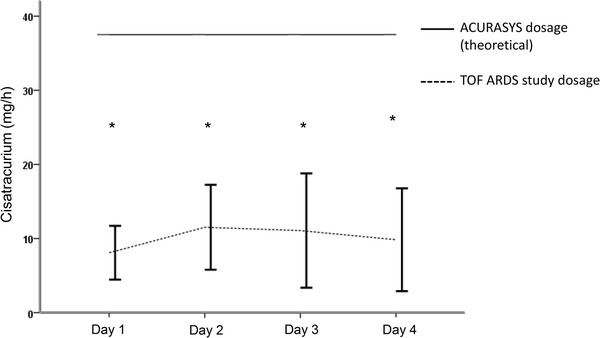
Mean daily cisatracurium dosage. The data are presented as the mean ± SD. The constant horizontal bar represents the theoretical dosage that would have been used following the ACURASYS study protocol (37.5 mg/h). *p < 0.001 between ACURASYS and TOF-ARDS dosage

The cisatracurium dose regimen significantly increased from the beginning to the end of the study (*p* = 0.003). Both initial and final dosages of cisatracurium were significantly lower than the dosage that would have been used according to the ACURASYS protocol, i.e., 37.5 mg per hour (*p* < 0.001) (Fig. 2).

**Fig. 3:**
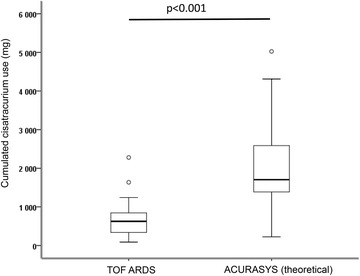
Cumulative cisatracurium doses received by the study patients compared to the theoretical dosage that would have been administered according to the ACURASYS study. The *box plot* limits represent the 25th and 75th percentiles, and the bars represent the 5th and 95th percentiles. The median is represented as a horizontal line. Extreme values are represented by *circles*

The overall consumption of NMBAs during the period of neuromuscular block was 700 ± 470 mg compared to 2040 ± 1119 mg if patients had received the ACURASYS dosage for the same period (*p* < 0.001) (Fig. 3).

### Neuromuscular block quality

**Fig. 4:**
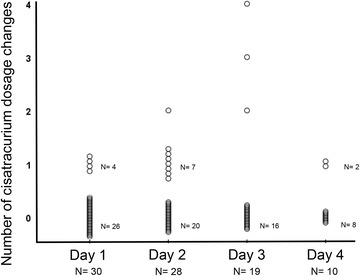
Cisatracurium dosage changes from day 1 to day 4. *Each circle* represents a patient. The total number of patients still receiving cisatracurium is given on the abscissa axis

The initial dose regimen of cisatracurium permitted to obtain 0 twitches at the first TOF (0/4) after the beginning of administration in 70% of cases. This dosage was the same from the beginning to the end of the study for 18 (60%) patients, whereas 12 patients needed a higher cisatracurium dose regimen. Only 4 patients (13%) had more than 1 dosage modification during the study period. Figure 4 represents the number of cisatracurium changes during the study period. Patient–ventilator asynchronies were clinically diagnosed requiring a cisatracurium rapid infusion and an increase in continuous posology in 4 (13%) patients. This occurred once for 3 patients and twice for 1 patient. Patient–ventilator asynchronies were observed soon after the initiation of cisatracurium infusion in 3 patients and at day 4 for the last one. However, pneumothorax or a decrease in SpO_2_ less than 85% was not reported.

### Increased cisatracurium consumption-associated factors

Factors associated with a cisatracurium dosage higher than 0.18 mg per kg per hour (initial posology) were analyzed. Only two parameters were statistically significantly associated with an increase in posology in a univariate analysis (Table 3): PEEP and driving pressure. Because of their collinearity, we performed two multivariate models (the first with PEEP at day 1 and the second with driving pressure at day 1) including all variables with a *p* value <0.2 in the univariate analysis (Table 4). In the first model, a lower PEEP was significantly associated with an increase in cisatracurium consumption, whereas in the second model, a higher driving pressure was linked to higher dosage of cisatracurium. The first model exhibited a better goodness of fit.

**Table 3:** Univariate analysis evaluating the factors associated with increase(s) in cisatracurium dosage

Variables	No cisatracurium dosage increase *N* = 18	Cisatracurium dosage increases *N* = 12	*p* value
Age, years	64 (47–70)	63 (53–76)	0.54
Female, *n* (%)	5 (28)	2 (17)	0.67
Height, cms	170 (160–175)	170 (161–174)	0.66
Weight, kgs	74 (59–86)	82 (68–110)	0.14
SAPS 2	47 (28–54)	44 (41–53)	0.78
SOFA	8 (7–12)	8 (7–10)	0.37
Characteristics at day 1			
Vt, mL	410 (380–420)	390 (360–437)	0.60
Vt, mL/kg (PBW)	6.2 (5.6–6.8)	6.2 (5.4–6.78)	0.85
Respiratory rate	26 (22–28)	24 (20–27)	0.25
PEEP, cm H_2_0	12 (10–14)	8 (8–11.5)	0.008
Pplat, cm H_2_0	25 (22–26)	25 (22–27)	0.92
Driving pressure, cm H_2_O	12 (10–15)	15 (13–16)	0.017
PaO_2_/FiO_2_ ratio	92 (70–119)	107 (100–121)	0.14
Severe versus moderate ARDS, *n* (%)	10 (55)	3 (25)	0.14
pH	7.32 (7.22–7.36)	7.33 (7.30–7.42)	0.49
PaCO_2_, mmHg	47 (39–57)	44 (41–57)	0.92
Prone position, *n* (%)	10 (55)	3 (25)	0.14
Bicarbonates, mmol/L	24 (21–29)	27 (24–32)	0.15
Lactate, mmol/L	1.28 (1.10–1.91)	1.27 (0.97–2.22)	0.87
Vasopressor use, *n* (%)	17 (94)	10 (83)	0.55
Norepinephrine, mg/h	1.1 (0.55–2.5)	1 (0.0–1.7)	0.32
Body temperature, °C	38.1 (37.5–38.6)	38.1 (37.4–38.6)	0.95
Ketamine use, *n* (%)	5 (28)	4 (33)	1
Other organ failure, (*n* 0/1/2/>2)	1/13/4/0	3/4/4/1	0.12

Variables are presented as the median ± interquartile range. *ARDS* acute respiratory distress syndrome, *PBW* predicted body weight, *PEEP* positive end-expiratory pressure, *Pplat*, end-inspiratory plateau pressure, *SAPS 2* Simplified Acute Severity Score 2, *SOFA*, Sepsis-related Organ Failure Assessment, *Vt* tidal volume, *ARDS* acute respiratory distress syndrome, *PEEP*, positive end-expiratory pressure

**Table 4:** Multivariate analysis evaluating the factors associated with increase(s) in cisatracurium dosage

Variables	Odds ratio	95% CI	*p* value
First model with PEEP at day 1			
Weight, per 1 kg increase	1.14	0.98–1.31	0.07
Bicarbonates, per 1 mmol/L	1.06	0.87–1.29	0.56
PEEP day 1, per 1 cm H_2_O increase	0.42	0.18–0.98	0.044
Severe ARDS	0.22	0.017–2.89	0.25
Use of prone position	1.73	0.14–22.06	0.67
Other organ failure, per organ	6.02	0.68–53.1	0.10
Second model with driving pressure at day 1			
Weight, per 1 kg increase	1.07	0.99–1.16	0.065
Bicarbonates, per 1 mmol/L	1.06	0.87–1.29	0.58
Driving pressure day 1, per 1 cm H_2_O increase	1.99	1.01–3.9	0.045
Severe ARDS	0.32	0.017–6.02	0.44
Use of prone position	6.98	0.32–153.4	0.22
Other organ failure, per organ	1.96	0.35–10.85	0.44

*ARDS* Acute Respiratory Distress Syndrome, *PEEP* positive end-expiratory pressure

### Cost benefits

We estimated that following our nurse-driven protocol, the mean cost-reduction linked to decreased cisatracurium consumption would be 70 euros/patient for the duration of treatment.

### Protocol deviations

The protocol for management of NMBAs was perfectly followed by nurses for 22 patients (73%). The main cause of inefficient use of the algorithm was excessive monitoring of TOF (every hour when not needed).

## Discussion

Our study originally demonstrated that a nurse protocol for NMBA management significantly reduced cisatracurium use during ARDS treatment without affecting the quality of the neuromuscular block in most patients. This study is the first to focus on nurse-managed administration of NMBAs in ARDS patients. When compared to the dosage used in 3 RCTs [3, 8, 20] showing a beneficial effect of NMBAs in ARDS patients, our nurse protocol based on TOF monitoring allowed a 60% reduction in the overall cisatracurium consumption. Moreover, in most cases, an effective neuromuscular block was obtained starting from the first hour, and modification of posology was not needed in 60% of patients.

In ARDS, the reduction in sedation posology and the algorithm-based management of sedation permitted a reduction in mortality [21]. In 2004, Baumann et al. [13] concluded that TOF monitoring had no effect either on NMBA consumption or on recovery time compared to clinical assessment. Similar results had been found in a study by Strange et al. [14] with atracurium. However, these studies did not focus on ARDS patients and employed a low dosage of cisatracurium that might not have been sufficient to ensure diaphragm paralysis. In a recently published work including a large cohort of ARDS patients [12], important discrepancies were found between the clinical appreciation of muscle paralysis and TOF monitoring. In this work, there was less than a 20% agreement between clinical judgment and TOF monitoring, regardless of the TOF site evaluation. Clinical judgment was not able to diagnose under- or over-paralysis compared to TOF monitoring. The most current recommendations consider associating TOF monitoring and clinical judgment [11].

The accurate posology of NMBAs to use during ARDS treatment is still unknown. High doses were used in the RCTs that showed a benefit to prognosis. A current study, trying to revaluate the role of cisatracurium during ARDS (ROSE study, NCT02509078), uses the same posology. Recent guidelines [11] recommend the use of continuous NMBA infusion in ARDS patients with a PaO_2_/FiO_2_ ratio under 150 but do not suggest any dosage. In a study by Bouju et al. [12], the cisatracurium dosage used was much lower than in the ACURASYS study. However, under-paralysis was frequently diagnosed by TOF, depending on the site of monitoring, including when voluntary efforts or patient-asynchrony was not diagnosed by clinicians. Using the same posology as Bouju et al., we observed that 40% of patients needed cisatracurium dose increases. However, the mean rise of the dose from the beginning to the end of the study was less than 20%. These results suggest that, even though the initial posology used in our protocol was probably insufficient to ensure a deep paralysis (when assessed on ulnar TOF monitoring), TOF monitoring and a relatively low increase in doses would allow for a rapid profound neuromuscular block. Regarding the quality of the muscle paralysis, lower total amounts of cisatracurium might be sufficient during ARDS treatment, provided that TOF is monitored.

The main advantage of a reduction in NMBA doses might be the reduction in the incidence of ICU-acquired weakness (ICUAW). Indeed, even though the use of a short course of cisatracurium has never been identified as an independent risk factor for ICUAW [22, 23], high doses and a long duration of treatment may favor ICUAW, especially when combined with glucocorticoids and/or sepsis [24–26]. Moreover, sparing the use of cisatracurium could represent an important cost-reduction.

We chose to monitor the TOF at the ulnar site. Important discrepancies have been demonstrated for muscle paralysis evaluation between ulnar and facial sites [12]. French recommendations have advised to preferentially use the facial site [27], whereas recent guidelines from the Society of Critical Medicine did not prioritize one site over the other [11]. Using the ulnar site, we chose to aim for a TOF goal of 0 twitches to ensure deep muscle paralysis and especially a diaphragmatic relaxation. Indeed, the *adductor pollicis* muscle is more sensitive to the action of NMBAs than the eyebrow muscle and the diaphragm [28–30].

Our work originally evaluated the feasibility of the management of NMBAs by nurses. Management of sedation and glycemic control [31] are already practiced in ICUs. In our work, nurses were able to perform the changes in posology as required by the protocol. Our results suggest that such an algorithm can be easily integrated into nurses’ responsibilities, provided that adequate training is provided. Boulila et al. [32] found similar results in the management of NMBAs during hypothermia after cardiac arrest.

Interestingly, a lower PEEP and a higher driving pressure were associated with the need of higher cisatracurium dosage. This suggests that an increase in lung stress and a decrease in thoraco-pulmonary compliance requested a more profound muscular paralysis. These results, however, deserve to be more precisely investigated.

Our study has several limitations. First, we did not use a control group but compared the doses of cisatracurium received to those that would have been used following the ACURASYS protocol. A further study, using a control group, would be necessary to confirm our results. Second, some patients were not immediately effectively paralyzed, even if very few patient-asynchronies occurred, suggesting that enhancing the initial dosage of cisatracurium may be required to obtain an earlier profound muscular paralysis. Finally, our study was designed to evaluate a nurse NMBA management protocol on the quality of the neuromuscular block. We cannot conclude on its effect on patient outcomes, even if protective ventilation was observed throughout the study. In particular, beneficial anti-inflammatory effects of NMBAs, as suggested in several studies [7, 8] could be reduced when decreasing the cisatracurium dose.

## Conclusion

The management of paralysis in patients ventilated for ARDS by nurses seems to be a feasible and secure procedure. It allows a drastic reduction in the cisatracurium dosage with a satisfying quality of muscle paralysis. A larger study using a randomized design that evaluates patient outcomes would permit confirmation of these results and help to develop a protocol for cisatracurium management and monitoring during ARDS treatment.

## Abbreviations

ARDS: acute respiratory distress syndrome
ECCO_2_R: extra-corporeal CO_2_ removal
ECMO: extra-corporeal membrane oxygenation
FiO_2_: fraction of inspired oxygen
ICU: intensive care unit
ICUAW: intensive care unit-acquired weakness
iNO: inhaled nitric oxide
MV: mechanical ventilation
NMBAs: neuromuscular-blocking agents
PaO_2_: arterial partial pressure of oxygen
PBW: predicted body weight
PEEP: positive end-expiratory pressure
Pplat: end-inspiratory plateau pressure
RASS: Richmond Agitation-Sedation Scale
RCTs: randomized controlled trials
SAPS 2: Simplified Acute Severity Score 2
SOFA: Sepsis-related Organ Failure Assessment
TOF: train-of-four
Vt: tidal volume
